# Household

**Published:** 1890-03

**Authors:** 


### HOUSEHOLD.

Burn Your Garbage.—The Seaside Sanitarium of New York City, has issued a circular letter of warning to housekeepers, the main points of which are—"Burn your garbage—put nothing in the ash barrel but ashes—don't delay—begin at once—Cholera and other epidemic diseases are bred in the ash barrels' decaying mixture," etc., etc. A timely warning which should not go unheeded.

Breakfast Cocoa.—In these days when food adulteration is so common, it is a comfort to find an article for the table that is thoroughly reliable. Walter Baker & Co.'s Breakfast Cocoa is eminent in this limited class. No chemicals are used in its manufacture, it is absolutely pure, and it is soluble. It forms, moreover, a delicious and healthful drink, as refreshing, and more nutritious than tea or coffee, and free from the injurious effects that those beverages sometimes produce. And it is very cheap withal. The house of Walter Baker & Co. has maintained for more than 100 years a great and honored repute by the excellence and purity of its manufactures.

Table of Measures and Weights.—Housekeepers will find a copy of this useful pasted over their baking-table :

4 Saltspoonfuls = 1 teaspoonful.
3 teaspoonfuls = 1 tablespoonful.
4 tablespoonfuls = ½ cup.
2 gills = 1 cup.
2 cups = 1 pint.
2 pints = 1 quart.
4 quarts = 1 gallon.
4 cups flour = 1 pound.
2 cups sugar = 1 "
3 cups meal = 1 "
1 cup solid butter = 1 pound.
1 heaping tablespoonful butter = 2 ounces.
1 " " sugar = 1 ounce.
1 tablespoonful liquid = ½ ounce.

Pruning Roses.—There are thousands of Rose bushes all over the country which, in spite of being found in spring to have made fine growth during the previous season, never produce good flowers, and the explanation is generally to be found in the fact that no reasonable plan is followed in pruning.

The commonest mistake is the leaving of the older branching spray wood that has already flowered. Dwarf Rose bushes at the beginning of the year generally consist of several much-branched stems which carried bloom in the previous summer, and several strong straight shoots springing from the base of the plant. In the case of Hybrid Perpetuals, these older branching stems should be cut completely out, leaving only the new shoots from the base which themselves should be then considerably shortened. Is the old spray would be left in it produces no flowers worth having, while the weak and crowded growths with which it becomes covered afford a perfect harborage to every known Rose pest.—*Vick's Magazine for* June.

